# Long-term variability in physiological measures in relation to mortality and epigenetic aging: prospective studies in the USA and China

**DOI:** 10.1186/s12916-022-02674-w

**Published:** 2023-01-16

**Authors:** Hui Chen, Tianjing Zhou, Shaowei Wu, Yaying Cao, Geng Zong, Changzheng Yuan

**Affiliations:** 1 School of Public Health, the Second Affiliated Hospital, Zhejiang University School of Medicine, Hangzhou, 310058 Zhejiang China; 2 Department of Occupational and Environmental Health, School of Public Health, Xi’an Jiaotong University Health Science Center, Xi’an, China; 3 CAS Key Laboratory of Nutrition, Metabolism and Food Safety, Shanghai Institute of Nutrition and Health, University of Chinese Academy of Sciences, Chinese Academy of Sciences, Shanghai, China; 4 The Key Laboratory of Intelligent Preventive Medicine of Zhejiang Province, Hangzhou, 310058 Zhejiang China; 5 Department of Nutrition, Harvard T.H. Chan School of Public Health, Boston, MA USA

**Keywords:** Body weight variability, Pulse rate variability, Blood pressure variability, Mortality, Epigenetic aging

## Abstract

**Background:**

Visit-to-visit body weight variability (BWV), pulse rate variability (PRV), and blood pressure variability (BPV) have been respectively linked to multiple health outcomes. The associations of the combination of long-term variability in physiological measures with mortality and epigenetic age acceleration (EAA) remain largely unknown.

**Methods:**

We constructed a composite score of physiological variability (0-3) of large variability in BWV, PRV, and BPV (the top tertiles) in 2006/2008–2014/2016 in the Health and Retirement Study (HRS) and 2011–2015 in the China Health and Retirement Longitudinal Study (CHARLS). All-cause mortality was documented through 2018. EAA was calculated using thirteen DNA methylation-based epigenetic clocks among 1047 participants in a substudy of the HRS. We assessed the relation of the composite score to the risk of mortality among 6566 participants in the HRS and 6906 participants in the CHARLS by Cox proportional models and then investigated its association with EAA using linear regression models.

**Results:**

A higher score of variability was associated with higher mortality risk in both cohorts (pooled hazard ratio [HR] per one-point increment, 1.27; 95% confidence interval [CI], 1.18, 1.39; *P*-heterogeneity = 0.344), after adjustment for multiple confounders and baseline physiological measures. Specifically, each SD increment in BWV, PRV, and BPV was related to 21% (95% CI: 15%, 28%), 6% (0%, 13%), and 12% (4%, 19%) higher hazard of mortality, respectively. The composite score was significantly related to EAA in second-generation clocks trained on health outcomes (e.g., standardized coefficient = 0.126 in the Levine clock, 95% CI: 0.055, 0.196) but not in most first-generation clocks trained on chronological age.

**Conclusions:**

Larger variability in physiological measures was associated with a higher risk of mortality and faster EAA.

**Supplementary Information:**

The online version contains supplementary material available at 10.1186/s12916-022-02674-w.

## Background

Global population aging forecasts an increasing burden of disease and disability on public health [1–3]. Aging is viewed as a gradual and progressive deterioration in the integrity of biological systems and can be indicated by molecular- and cellular-level changes [4]. Specifically, the loss of ability in homeostasis maintenance is a key feature of biological aging [5], potentially through the hypothalamus-related regulatory axes [6].

Older adults tend to have fluctuating physiological measures [7, 8], and previous studies have linked the long-term variabilities in these measures to subsequent risk of multiple aging-related health outcomes, including frailty [9, 10], cardiovascular diseases [11, 12], dementia [13–15], and mortality [16]. For example, a meta-analysis [16] showed that each SD increment in BPV was associated with a 15% higher risk of all-cause mortality. However, the overall associations of long-term variabilities in major physiological measures with aging remained unclear. First, most previous studies focused on one specific type of physiological measure, while aging is often accompanied by homeostatic imbalances in multiple systems [4], and the combined relation of these variability measures to mortality warrants investigation across populations. Second, most previous studies utilized relatively short-term measurements of physiological measures, and the relation of long-term variability in physiological measures with mortality has been less explored. Moreover, the underlying mechanism remained unclear. Although epigenetic clocks have been suggested to capture the complexity between aging and homeostasis maintenance [17] and reflect biological aging [18–21], few studies have examined their associations with variabilities in physiological measures.

Therefore, we hypothesize that the long-term variability in multiple physiological measures could be linked to accelerated aging. To assess the relation of the combination of BWV, PRV, and BPV with mortality and epigenetic accelerated aging (EAA), we leveraged two nationally representative prospective studies, the Health and Retirement Study (HRS) in the USA and the China Health and Retirement Longitudinal Study (CHARLS). In addition, the HRS 2016 Venous Blood Study (VBS) provided a unique opportunity to analyze a panel of well-constructed epigenetic clocks based on high-quality DNA methylation data [22].

## Methods

### Study design and population

This study was based on the Health and Retirement Study (HRS) [23] and the China Health and Retirement Longitudinal Study (CHARLS) [24], two national cohort studies with similar designs [25, 26].

In the HRS, participants aged 50 or older were recruited and revisited biennially for collection of sociodemographic, lifestyle, and clinical information. Half of a randomly selected sample received physical examinations in 2006, 2010, and 2014 and the other half in 2008, 2012, and 2016 [25]. DNA methylation assays were conducted in a representative subsample of the HRS 2016 Venous Blood Study (VBS) with comparable distributions of age, sex, education, and race/ethnicity to the entire HRS 2016 sample born before 1960 [27]. For example, the HRS 2016 sample had a mean age of 69.0 years and 59.3% female, and the corresponding values were 68.7 years and 58.0% in the VBS. The HRS was approved by the Health Sciences and Behavioral Sciences Institutional Review Board at the University of Michigan (HUM00061128).

The CHARLS recruited participants aged 45 years or older in 2011 and revisited them in 2013, 2015, and 2018. Face-to-face interviews and physical examinations were administered to all participants at each wave. The CHARLS was approved by the Ethics Committee of Pecking University (00001052-11, 014).

**Fig. 1:**
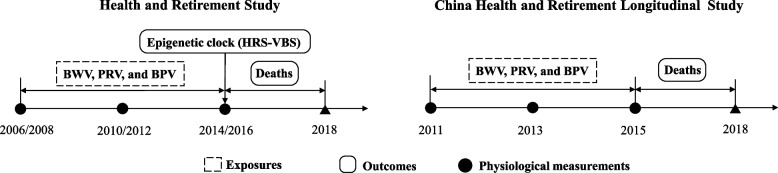
Study design. BWV, body weight variability; PRV, pulse rate variability; BPV, blood pressure variability; BMI, body mass index

We conducted two primary analyses (Fig. 1 and Additional file 1: Fig. S1). First, we assessed the relations of a composite score of variability in physiological measures to mortality, including 6566 HRS participants and 6906 CHARLS participants who (1) received three measurements of body mass index (BMI), pulse rate, and blood pressure in 2006–2016 (HRS) or 2011–2015 (CHARLS) and (2) were followed up to the next wave or had death information during follow-up. Among the HRS-VBS participants who had high-quality DNA methylation data in 2016 (*N* = 1047), we investigated the association between the composite score and EAA.

### Variability in body weight, pulse rate, blood pressure, and composite score

Body weight, height, pulse rate, and blood pressure in the HRS (2006–2014 or 2008–2016) and the CHARLS (2011–2015) were measured following similar protocols. Body weight was measured using an electronic weighing scale without shoes and with light clothing, and height was measured by having the respondent stand against a wall without shoes [28, 29]. BMI was calculated as weight in kilograms divided by height in meters squared. Pulse rate and blood pressure were each assessed three times, 45 to 60 s apart, respectively, in a seated position, by trained interviewers using an automated sphygmomanometer [28, 30]. The average values of the last two valid measurements were used at each wave.

Visit-to-visit BWV, PRV, and BPV were calculated as the coefficients of variation (CV), i.e., quotient of standard deviation (SD) and mean, of BMI, pulse rate, and systolic blood pressure (SBP), respectively. We used systolic blood pressure variability (SBPV) to represent BPV because it had stronger associations with adverse health outcomes than diastolic blood pressure (DBP) [31, 32]. We also used DBPV as an alternative BPV measure in the sensitivity analysis.

To reflect the overall variability in physiological measures, we constructed a composite score following the strategy of a prior study [33]. The highest tertiles of BWV, PRV, and BPV were each assigned 1 point, and the middle and lowest tertiles were assigned 0 point. We took the sum of the three binary indicators as the composite score (range: 0–3), and a higher score represented greater intraindividual instability in the physiological measures.

### Ascertainment of mortality

All-cause mortality was documented from the latest physiological measurements (2014 or 2016 in HRS and 2015 in CHARLS) to the end of follow-up (2018). The HRS and CHARLS adopted similar strategies to identify deaths using data linkage from the population registry and through interviews with informants or knowledgeable others [34, 35].

### Epigenetic clock and epigenetic age acceleration

In the HRS-VBS, DNA methylation was assayed using the Infinium Methylation EPIC BeadChip that covered over 850,000 CpG sites (test-retest reliability correlation > 0.97 for all CpG sites) [36]. Thirteen epigenetic clocks were constructed based on DNA methylation data. Nine first-generation clocks (Horvath 1, Hannum, Horvath 2, Lin, Weidner, VidalBralo, Yang, Bocklandt, and Garagnani) were trained on chronological age, and four second-generation clocks (Levine, Zhang, GrimAge, and DunedinPoAm38) were trained on health-related outcomes [22, 37]. The thirteen epigenetic clocks [38–50] combined information for a number of CpGs (typically 100–500) to produce indicators of epigenetic aging. For example, Horvath 1 used 353 CpGs selected by the elastic net regression model [38]. Missing beta methylation values were imputed with the mean value of the given probe across all samples [36]. More information can be found in the HRS-VBS protocol [36].

We defined EAA following the strategy in a previous study in the HRS-VBS [22]: each clock value was regressed on chronological age in 2016 and the standardized residual reflected EAA. A larger value of the standardized residual indicated faster biological aging when the chronological age was fixed [51].

### Other covariates

In HRS and CHARLS, demographic, lifestyle, and clinical factors were collected using questionnaires at baseline, including age, gender defined by self-identity, education level (high school degree level, yes/no), marital status (married or not), smoking status (never/former/current), alcohol consumption (never/former/current), household income (in quartiles for HRS and < 9999 yuan/≥ 10,000 yuan/missing for CHARLS), physical activity engagement (> 1 time per week/1–3 times per month/never for HRS and any/never/missing for CHARLS), and self-reported diabetes mellitus, heart disease, and stroke. In the HRS, race (white/black/others) was self-reported. In the CHARLS, residence (rural/urban) was identified from the registry.

### Statistical analyses

Baseline characteristics of the 6566 HRS participants, 6906 CHARLS participants, and 1047 HRS-VBS participants were presented in mean ± standard deviation (SD) for continuous variables and number (percentage) for categorical variables.

In the primary analyses, we assessed the association between the composite score of variability in physiological measures and risk of mortality using Cox proportional hazard models. Person-time was calculated from the latest physiological measurement to the year of death or the end of follow-up, whichever came first. We sequentially adjusted the hazard ratios (HRs) and 95% confidence intervals (CIs) for baseline age, gender, race (for HRS only), residence (for CHARLS only), education level, household income, marital status, smoking status, alcohol consumption, physical activity engagement, baseline BMI, baseline pulse rate, and baseline blood pressure. The estimates from the two cohorts were pooled using fixed-effect models when no significant heterogeneity was detected. Missing values for all continuous covariates (< 2%) were imputed by means and categorical variables by the most populated categories, except for household income and physical activity engagement in the CHARLS (using a missing category). We used linear regression models to evaluate the associations between the composite score and EAA indicated by the 13 epigenetic clocks in the HRS. Linear trend was tested by assigning the median value of each category and including it as a continuous variable in the models. To address multiplicity, we adjusted the *P*-values using the Benjamini-Hochberg (B-H) methods in the analysis of the composite score and EAA [52].

In the secondary analysis, we assessed the individual associations of BWV, PRV, and BPV with mortality and EAA, respectively. The adjustment was the same as the primary analysis, and baseline BMI, baseline pulse rate, and baseline blood pressure were separately adjusted for the corresponding exposures.

We performed multiple sensitivity analyses by (1) adjusting the models for baseline health conditions (diabetes mellitus, heart disease, and stroke); (2) alternatively using standard deviation (SD) and variation independent of the mean (VIM) to represent BWV, PRV, and BPV; (3) using DBPV in place of SBPV; (4) constructing the composite score using the medians of BWV, PRV, and BPV as cutoffs rather than the top tertiles; (5) alternatively assigning 0, 1, and 2 to the increasing tertiles of variability indicators to construct a new composite score ranging from 0 to 6 and repeated the primary analysis; and (6) adjusting the models for cell-type proportions (the percentages of neutrophil, basophilic granulocyte, eosinophilic granulocyte, mononuclear granulocyte, and leukomonocyte in the total count of white blood cells) in the analysis of EAA [53, 54].

Statistical analyses were performed using R 4.1.0. We reported two-sided *P* values and 95% confidence intervals (CIs) throughout, and tests with *P* < 0.05 were considered statistically significant.

## Results

### Participant characteristics at baseline

Baseline characteristics of the study population in the HRS cohort (*N* = 6566), HRS-VBS (*N* = 1047), and CHARLS (*N* = 6906) were presented in Table 1. Compared with HRS participants, adults in the CHARLS were younger (62.5 years vs. 73.0 years), fewer in females (54.2% vs. 60.7%), less educated, more likely to be current smokers, less likely to be current alcohol consumers, and had smaller BWV, PRV, and BPV. Among the 1047 HRS-VBS participants, 59.6% were female, and 87.3% were White/Caucasian. The mean (SD) age of the HRS-VBS participants was 77.0 (5.5) years old, and the means of long-term BWV, PRV, and BPV were 4.9%, 9.0%, and 9.3%, respectively. Descriptive statistics of the 13 epigenetic clocks and EAA were displayed in Additional file 1: Table S1.

**Table 1:** Baseline characteristics of the study participants

Characteristics^**a**^	HRS		CHARLS
	HRS cohort	HRS-VBS	
*N*	6566	1047	6906
Age, years, mean ± SD	73.0 ± 9.1	77.0 ± 5.5	62.5 ± 9.0
Female, *N* (%)	3988 (60.7)	624 (59.6)	3741 (54.2)
Having high school degree level, *N* (%)	5594 (85.2)	881 (84.1)	633 (9.2)
Married, *N* (%)	4093 (62.4)	652 (62.3)	5648 (81.8)
Caucasian, *N* (%)	5420 (82.5)	914 (87.3)	–
Rural resident, *N* (%)	–	–	4792 (69.4)
Smoking status, *N* (%)			
Current	602 (9.2)	68 (6.5)	1849 (26.8)
Former	2944 (45.0)	519 (49.6)	1148 (16.6)
Never	3013 (46.2)	460 (43.9)	3909 (56.6)
Alcohol consumption, *N* (%)			
Current	2522 (38.5)	382 (36.5)	2220 (32.2)
Former	1080 (16.5)	151 (14.4)	817 (11.8)
Never	2944 (45.0)	514 (49.1)	3869 (56.0)
Stroke, *N* (%)	609 (9.3)	118 (11.3)	263 (3.8)
Heart diseases, *N* (%)	1963 (30.0)	379 (36.2)	1230 (18.2)
Diabetes, *N* (%)	1655 (25.2)	299 (28.6)	679 (10.1)
Body mass index, kg/m^2^, mean ± SD	29.2 ± 5.6	29.0 ± 5.6	23.7 ± 4.2
Systolic blood pressure, mmHg, mean ± SD	129.3 ± 19.1	130.1 ± 20.0	128.0 ± 20.2
Pulse rate, bpm, mean ± SD	68.8 ± 10.9	67.8 ± 10.6	73.9 ± 10.8
BWV, %, mean ± SD	5.3 ± 4.6	4.9 ± 4.4	4.2 ± 5.2
BPV, %, mean ± SD	9.3 ± 5.5	9.3 ± 5.3	8.7 ± 5.6
PRV, %, mean ± SD	9.1 ± 5.8	9.0 ± 5.8	8.9 ± 5.2
Household income, *N* (%)			
Quartile 1	1643 (25.0)	262 (25.0)	–
Quartile 2	1642 (25.0)	262 (25.0)	–
Quartile 3	1640 (25.0)	261 (25.0)	–
Quartile 4	1641 (25.0)	262 (25.0)	–
0–9999 yuan	–	–	1909 (27.6)
≥ 10,000 yuan	–	–	2265 (32.8)
Missing	–	–	2732 (39.6)
Physical activity, *N* (%)			
≥ 1 time/week	2305 (35.1)	350 (33.4)	–
< 1 time/week	3941 (60.1)	647 (61.8)	–
Never	314 (4.8)	50 (4.8)	–
Any	–	–	1922 (27.8)
Never	–	–	1499 (21.7)
Missing	–	–	3485 (59.5)

*HRS* Health and Retirement Study, *CHARLS* China Health and Retirement Longitudinal Study, *HRS-VBS* Health and Retirement Study - Venous Blood Study, *BMI* body mass index, *Q* quartile, *BWV* body weight variability, *BPV* blood pressure variability, *PRV* pulse rate variability

^a^Values are described as mean ± SD and percentage (%) for continuous and categorical variables, respectively

### Associations between the composite score of variability and mortality and epigenetic age acceleration

**Fig. 2:**
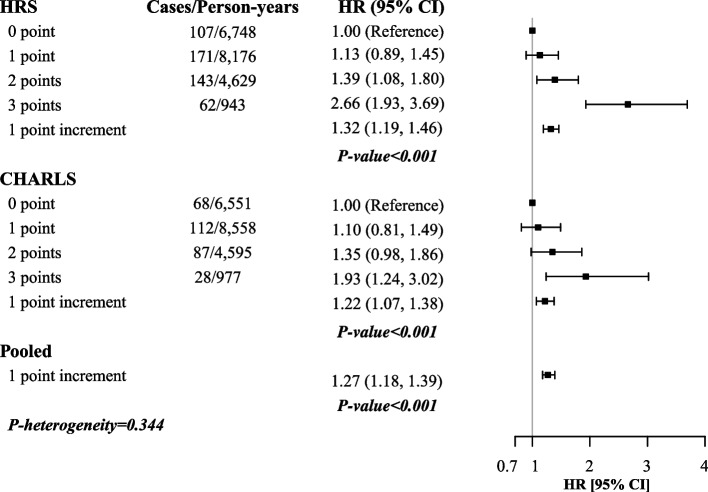
Multivariable adjusted hazard ratios (HRs) and 95% confidence intervals (CIs) of mortality associated with the composite score of variability in the HRS and CHARLS. HRS, Health and Retirement Study; CHARLS, China Health and Retirement Longitudinal Study; HR, hazard ratio; CI, confidence interval. Models were adjusted for age (continuous), gender (female/male), education (high school degree level, yes/no), marriage status (yes/no), residence (rural/urban) in the CHARLS, race (White/Black/others) in the HRS, drinking status (current/ever/never), smoking status (current/ever/never), physical activity (> 1 time per week/1–3 times per month/never in the HRS and any/never/missing in the CHARLS), household income (quartiles in the HRS and ≤ 9999 yuan/≥ 10,000 yuan/missing in the CHARLS), body mass index, systolic blood pressure, and pulse rate (all continuous) in 2014/2016 (HRS) or in 2015 (CHARLS). Fixed effect model was used to pool the results

A total of 778 participants died during follow-up, including 483 of the 6566 HRS participants (20,496 person-years) and 295 of the 6906 CHARLS participants (20,681 person-years). The association between the composite score of variability in physiological measures and mortality was observed in both cohorts (Fig. 2). Adjusted for multiple confounders and baseline levels of these physiological measures, each point increment of the composite score was related to a 27% (95% CI: 18%, 39%, *P*-heterogeneity = 0.344) higher risk of mortality. The corresponding HRs (95% CIs) were 1.32 (1.19, 1.46) in the HRS and 1.22 (1.07, 1.38) in the CHARLS. Compared with a variability score of 0, a score of 3 (co-existence of large BWV, PRV, and BPV) was associated with the highest risk of mortality (HR = 2.66, 95% CI: 1.93, 3.69 in the HRS and HR = 1.93, 95% CI: 1.24, 3.02 in the CHARLS). Detailed results could be found in Additional file 1: Table S2.

**Fig. 3:**
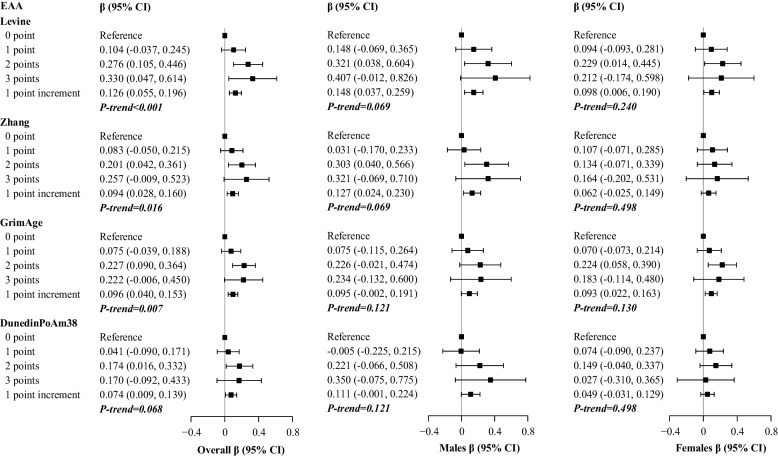
Multivariable adjusted differences and 95% confidence intervals (CIs) in epigenetic age acceleration indicated by second-generation clocks associated with the composite score of variability in the overall population and by gender. EAA, epigenetic age acceleration. β-Coefficients were derived from models adjusted for age (continuous), gender (female or male, unless in gender-stratified analyses), education (high school degree level, yes/no), marriage status (yes/no), race (White/Black/others), drinking status (current/ever/never), smoking status (current/ever/never), physical activity (> 1 time per week/1–3 times per month/never), household income (in quartiles), body mass index, systolic blood pressure, and pulse rate (all continuous) in 2014/2016. *P*-values were adjusted using the Benjamini-Hochberg methods

Also, a greater composite score of variability was associated with faster EAA (*z*-score of the regression residual of the epigenetic clock on chronological age) in four second-generation clocks trained on health outcomes, and the associations were consistently observed in both male and female participants (Fig. 3). Each point increment in the score was related to faster EAA by 0.126 *z*-score (95% CI: 0.055, 0.196) in the Levine clock, 0.094 *z*-score (95% CI: 0.028, 0.160) in the Zhang clock, 0.096 *z*-score (95% CI: 0.040, 0.153) in the GrimAge clock, and 0.074 *z*-score (95% CI: 0.009, 0.139) in DunedinPoAm38. All tests were significant after B-H adjustments (adjusted *P*-values < 0.05) except for that of DunedinPoAm38 (adjusted *P*-value = 0.068). The relations were non-significant in most first-generation clocks that are trained on chronological age, although the direction was similar. For example, the standardized coefficient (95% CI) was 0.051 (− 0.020, 0.122) for the Horvath 1 clock. Details were displayed in Additional file 1: Table S3.

### Associations of BWV, PRV, and BPV with mortality and epigenetic age acceleration

We observed non-heterogeneous associations of BWV, PRV, and BPV with mortality in both HRS and CHARLS (*P*-heterogeneity > 0.05, Table 2). The pooled HRs (95% CIs) of mortality for each SD increment in BWV, PRV, and BPV were 1.21 (95% CI: 1.15, 1.28), 1.06 (1.00, 1.13), and 1.12 (1.04, 1.19), respectively. The corresponding HRs (95% CIs) were 1.24 (1.15, 1.33), 1.07 (0.99, 1.16), 1.11 (1.03, 1.21) in the HRS and 1.17 (1.07, 1.29), 1.05 (0.95, 1.17), 1.12 (1.01, 1.24) in the CHARLS.

**Table 2:** Multivariable adjusted hazard ratios (HRs)^a^ and 95% confidence intervals (CIs) of mortality associated with body weight variability, pulse rate variability, and blood pressure variability in the HRS and CHARLS

Variable	HRS			CHARLS			Pooled HR^**b**^	***P***-heterogeneity
	Median, %	Cases/person-years	HR (95% CI)	Median, %	Cases/person-years	HR (95% CI)		
**Body weight variability**								
Tertile 1	1.9	124/6934	1.00 (reference)	1.5	79/6894	1.00 (reference)	1.00 (reference)	/
Tertile 2	4.0	129/6900	1.01 (0.78, 1.30)	3.1	85/6895	1.16 (0.85, 1.58)	1.07 (0.88, 1.30)	0.499
Tertile 3	8.0	230/6662	**1.67 (1.33, 2.11)**	6.0	131/6892	**1.57 (1.18, 2.09)**	**1.63 (1.36, 1.95)**	0.742
Per SD increment			**1.24 (1.15, 1.33)**			**1.17 (1.07, 1.29)**	**1.21 (1.15, 1.28)**	0.336
*P*-value			**< 0.001**			**0.001**		
**Pulse rate variability**								
Tertile 1	3.9	148/6841	1.00 (reference)	3.8	77/6898	1.00 (reference)	1.00 (reference)	/
Tertile 2	8.0	134/6830	0.86 (0.68, 1.09)	7.6	95/6894	1.07 (0.79, 1.45)	0.93 (0.78, 1.13)	0.265
Tertile 3	14.3	201/6825	1.13 (0.91, 1.41)	13.3	123/6889	1.17 (0.87, 1.57)	1.14 (0.96, 1.36)	0.853
Per SD increment			1.07 (0.99, 1.16)			1.05 (0.95, 1.17)	1.06 (1.00, 1.13)	0.777
*P*-value			0.132			0.341		
**Blood pressure variability**								
Tertile 1	4.2	131/6787	1.00 (reference)	4.0	87/6894	1.00 (reference)	1.00 (reference)	/
Tertile 2	8.2	140/6933	0.98 (0.77, 1.25)	8.0	92/6889	0.93 (0.69, 1.25)	0.96 (0.79, 1.16)	0.789
Tertile 3	14.4	212/6776	1.20 (0.96, 1.50)	13.6	116/6898	1.14 (0.86, 1.51)	1.17 (0.99, 1.40)	0.780
Per SD increment			**1.11 (1.03, 1.21)**			**1.12 (1.01, 1.24)**	**1.12 (1.04, 1.19)**	0.893
*P*-value			0.073			**0.036**		

*HRS* Health and Retirement Study, *CHARLS* China Health and Retirement Longitudinal Study, *SD* standard deviation

^a^Hazard ratios were adjusted for age (continuous), gender (female/male), education (high school degree level, yes/no), marriage status (yes/no), residence (rural/urban) in the CHARLS, race (White/Black/others) in the HRS, drinking status (current/ever/never), smoking status (current/ever/never), physical activity (> 1 time per week/1–3 times per month/never in the HRS and any/never/missing in the CHARLS), household income (quartiles in the HRS and ≤ 9999 yuan/≥ 10000 yuan/missing in the CHARLS), and body mass index/systolic blood pressure/pulse rate (all continuous) in 2014/2016 (HRS) or in 2015 (CHARLS)

^b^HRs were pooled using fixed effect models

In the HRS-VBS, the associations of BWV, PRV, and BPV with EAA varied across epigenetic clocks (Additional file 1: Fig. S2 and Table S4). Larger PRV was related to significantly faster EAA in GrimAge (*β* for per SD increment = 0.055 *z*-score, 95% CI: 0.005, 0.104). Larger BPV was associated with significantly faster EAA in Zhang (0.079 *z*-score, 95% CI: 0.020, 0.138) and Horvath 1 (0.084 *z*-score, 95% CI: 0.020, 0.147). For other non-significant associations, the directions were generally consistent with our hypotheses.

### Sensitivity analyses

The results in the sensitivity analyses were consistent with the primary findings (Additional file 1: Tables S5 and S6). When further adjusted for major health conditions, the relations of the composite score of variability to mortality and EAA persisted. When SD and VIM were used to measure the variability in physiological measures, the associations were comparable to the primary findings. When we replaced SBPV with DBPV, the relations were generally unchanged. When we redefined the cutoffs for large variability as medians or alternatively assigned 0, 1, and 2 to the increasing tertiles of variability indicators, the relations of the composite score to mortality and EAA remained similar. When further adjusted for cell-type proportions in the analysis of EAA, the relations were generally the same.

## Discussion

In two independent large cohorts of middle-aged and older adults in the USA and China, a higher composite score of long-term variability in body weight, pulse rate, and blood pressure was related to a higher risk of mortality independent of their baseline levels. Also, a higher score of variability was associated with faster EAA in all four second-generation epigenetic clocks that are trained on health outcomes. Aggregately, our findings suggest that instability in major physiological measures might be an important predictor of mortality and an indicator of biological aging in middle-aged and older adults.

The current study is one of the few that has assessed the relations of variabilities in physiological measures to mortality. In previous studies, BWV [5], PRV [55], and BPV [16] have been associated with mortality separately. For example, in the Framingham Heart Study, the highest degree of BWV was related to a higher risk (27% in men and 65% in women) of mortality as compared with those with the lowest BWV, which was confirmed by our analysis [5]. Although the individual associations of the variabilities in physiological measures with health outcomes have been widely explored [5, 33, 55, 56], little is known about their combined association with health outcomes. In a cohort study of Korean adults, larger 13-year variability in blood pressure, glucose, cholesterol, and BMI were related to higher mortality risk in the subsequent 3 years, and the HR (95% CI) for mortality was 1.21 (1.18, 1.23) for each unit increment in the score (range: 0–4) combining these indicators [33]. In our study, the combined and individual associations of 8-year (in HRS) or 4-year (in CHARLS) variability in physiological measures with mortality were consistently strong in two nationally representative cohorts in the USA and China, and the associations were independent of the baseline levels of these physiological measures. Our findings suggested that a composite score constructed with long-term variability indicators in non-invasive measurements may be a novel predictor or risk factor for mortality. However, whether the composite score is associated with long-term risk of mortality and whether the associations are causal warrant further investigation.

To our knowledge, this is the first study relating long-term variability in physiological measures to epigenetic aging. Previous studies have suggested that physiological dysfunction might be a result of epigenomic modifications [40], but the association between them was far from conclusive [57]. Our findings added valuable evidence to the current literature that long-term variability in physiological measures could be predictors of EAA, which implied a potential link between physiological homeostasis and DNA methylation [58]. Moreover, we found that the relation was more significant in the second-generation clocks than the first-generation clocks, potentially because the second-generation clocks are more relevant to aging-related health outcomes than the first-generation clocks. Although the underlying biological pathways remain largely unknown, previous studies have suggested a role for methylation in the crosstalk between the adaptive immune system and physiological homeostasis [59]. Also, global DNA methylation might also reflect fat distribution and homeostasis of certain biomarkers, although the direction of the causal link has yet to be confirmed [59]. Overall, by incorporating EAA, a reliable estimation of biological aging [40], our findings highlight the ability of physiological variability to reflect the pace of biological aging. Our findings that the observed association was independent of baseline levels of physiological status may suggest that longitudinal dynamic physiological statuses are important additions to their static values in the assessment of the pace of aging.

Several possible pathways could explain the observed associations, including cardiac and vascular regulation and inflammation, which were related to mortality and biological aging, respectively. For example, BPV and PRV were associated with poor cardiac and vascular regulation [60, 61], and BWV was linked to adipose tissue inflammation [62] and adipokine secretion alteration [63, 64]. Furthermore, the disturbance of homeostasis is often accompanied by inflammation [65], which may be linked to DNA methylation and demethylation processes [66]. However, current evidence is not sufficient for the assertion of their causal relation, as vascular and metabolic dysfunction [67–70] could be the common reasons for the loss of homeostasis and faster aging. More experimental and observational studies are required to further reveal the complex relations and underlying mechanisms.

The strengths of the present study included the two nationally representative cohorts, the high rates of follow-up, the objectively measured exposures, and the relatively large sample sizes. The high-quality epigenetic clock data also enabled us to further assess the association from a novel perspective. Nevertheless, our findings should be interpreted with caution due to some limitations. First, our findings did not necessarily suggest a causal link between the variability indicators and mortality or EAA, as residual confounding might still exist. For example, we did not fully account for intentional physiological changes, and physiological instability due to underlying diseases could not be fully eliminated. Second, the follow-up duration for mortality was relatively short, and future studies are needed to investigate the association of variability in physiological measures with mortality over a longer term. Third, we only included participants who attended multiple physical examinations, which limited the generalizability of our findings as they might be healthier than the general population. Moreover, while the indicators were significantly related to EAA, the overall variation explained was relatively low in most clocks (< 2% in all clocks, data not shown). Although previous studies also reported a low variance explained when assessing some well-established risk factors of aging, this might reflect the measurement errors in DNA methylation. Lastly, the generalizability of our findings on EAA should be validated in other populations, especially given that DNA methylation may differ across races and regions [71].

## Conclusions

Our prospective study suggests that a higher composite score of long-term variability in physiological measures, including body weight, pulse rate, and blood pressure, is related to an increased risk of mortality and faster epigenetic age acceleration independent of their baseline levels. Our findings underscored the potential role of homeostasis maintenance in the aging process. Future research is needed to confirm the study findings and to explore the underlying biological mechanisms.

## Supplementary Information

**Additional file 1: Fig. S1.** Participant inclusion and exclusion flowchart. **Fig. S2.** Scatter plots of BWV, PRV, and BPV with epigenetic age acceleration indicated by 4 second-generation epigenetic clocks. **Table S1.** Descriptive statistics of the 13 epigenetic clocks (N=1,047). **Table S2.** Associations of the composite score of variability in physiological measures with mortality in the HRS and CHARLS with different adjustments. **Table S3.** Associations between each one-point increment in the composite score of variability and epigenetic age acceleration indicated by 13 first- and second-generation epigenetic clocks. **Table S4.** Associations of BWV, PRV and SBPV with epigenetic age acceleration indicated by 13 first- and second-generation epigenetic clocks. **Table S5.** Sensitivity analyses for the associations between each one-point increment in the composite score of variability with risk of all-cause mortality. **Table S6.** Sensitivity analyses for the associations between variability of physiological measures and epigenetic age acceleration.

## Data Availability

The datasets that support the findings of this study are available on the HRS website (https://hrsonline.isr.umich.edu/) [23] and the CHARLS website (http://charls.pku.edu.cn/en/) [24].

## Abbreviations

BMI: Body mass index
BPV: Blood pressure variability
BWV: Body weight variability
CHARLS: China Health and Retirement Longitudinal Study
CI: Confidence interval
CV: Coefficient of variation
DBPV: Diastolic blood pressure variability
EAA: Epigenetic age acceleration
HR: Hazard ratio
HRS: Health and Retirement Study
PRV: Pulse rate variability
SBPV: Systolic blood pressure variability
SD: Standard deviation
VBS: Venous Blood Study
