# Supplementary material for: Long-term variability in physiological measures in relation to mortality and epigenetic aging: prospective studies in the USA and China
Source: BMC Med. 2023 Jan 16;21:20. doi: 10.1186/s12916-022-02674-w (PMC9843964; doi:10.1186/s12916-022-02674-w)
Supplement: Supplementary file 1 — Additional file 1: Fig. S1. Participant inclusion and exclusion flowchart. Fig. S2. Scatter plots of BWV, PRV, and BPV with epigenetic age acceleration indicated by 4 second-generation epigenetic clocks. Table S1. Descriptive statistics of the 13 epigenetic clocks (N=1,047). Table S2. Associations of the composite score of variability in physiological measures with mortality in the HRS and CHARLS with different adjustments. Table S3. Associations between each one-point increment in the composite score of variability and epigenetic age acceleration indicated by 13 first- and second-generation epigenetic clocks. Table S4. Associations of BWV, PRV and SBPV with epigenetic age acceleration indicated by 13 first- and second-generation epigenetic clocks. Table S5. Sensitivity analyses for the associations between each one-point increment in the composite score of variability with risk of all-cause mortality. Table S6. Sensitivity analyses for the associations between variability of physiological measures and epigenetic age acceleration. [file 12916_2022_2674_MOESM1_ESM.docx]

**Additional file 1**

**Supplementary Figures and Tables**

**Contents**

[Fig. S1. Participant inclusion and exclusion flowchart 2](#_Toc119529831)

[Fig. S2. Scatter plots of BWV, PRV, and BPV with epigenetic age acceleration indicated by 4 second-generation epigenetic clocks 3](#_Toc119529832)

[Table S1. Descriptive statistics of 13 epigenetic clock (N=1,047). 4](#_Toc119529833)

[Table S2. Associations of the composite score of variability in physiological measures with mortality in the HRS and CHARLS with different adjustments. 5](#_Toc119529834)

[Table S3. Associations between each one-point increment in the composite score of variability and epigenetic age acceleration indicated by 13 first- and second-generation epigenetic clocks. 6](#_Toc119529835)

[Table S4. Associations of BWV, PRV and SBPV with epigenetic age acceleration indicated by 13 first- and second-generation epigenetic clocks. 7](#_Toc119529836)

[Table S5. Sensitivity analyses for the associations between each one-point increment in the composite score of variability with risk of all-cause mortality. 9](#_Toc119529837)

[Table S6. Sensitivity analyses for the associations between variability of physiological measures and epigenetic age acceleration 10](#_Toc119529838)

# Fig. S1. Participant inclusion and exclusion flowchart


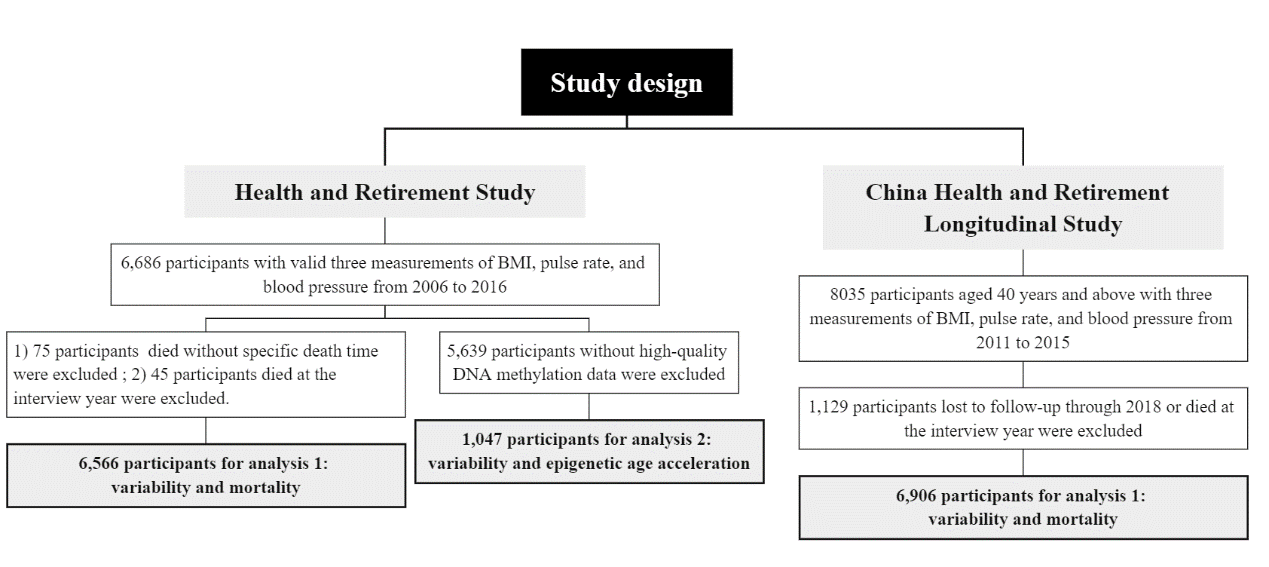


BMI: body mass index

# Fig. S2. Scatter plots of BWV, PRV, and BPV with epigenetic age acceleration indicated by 4 second-generation epigenetic clocks


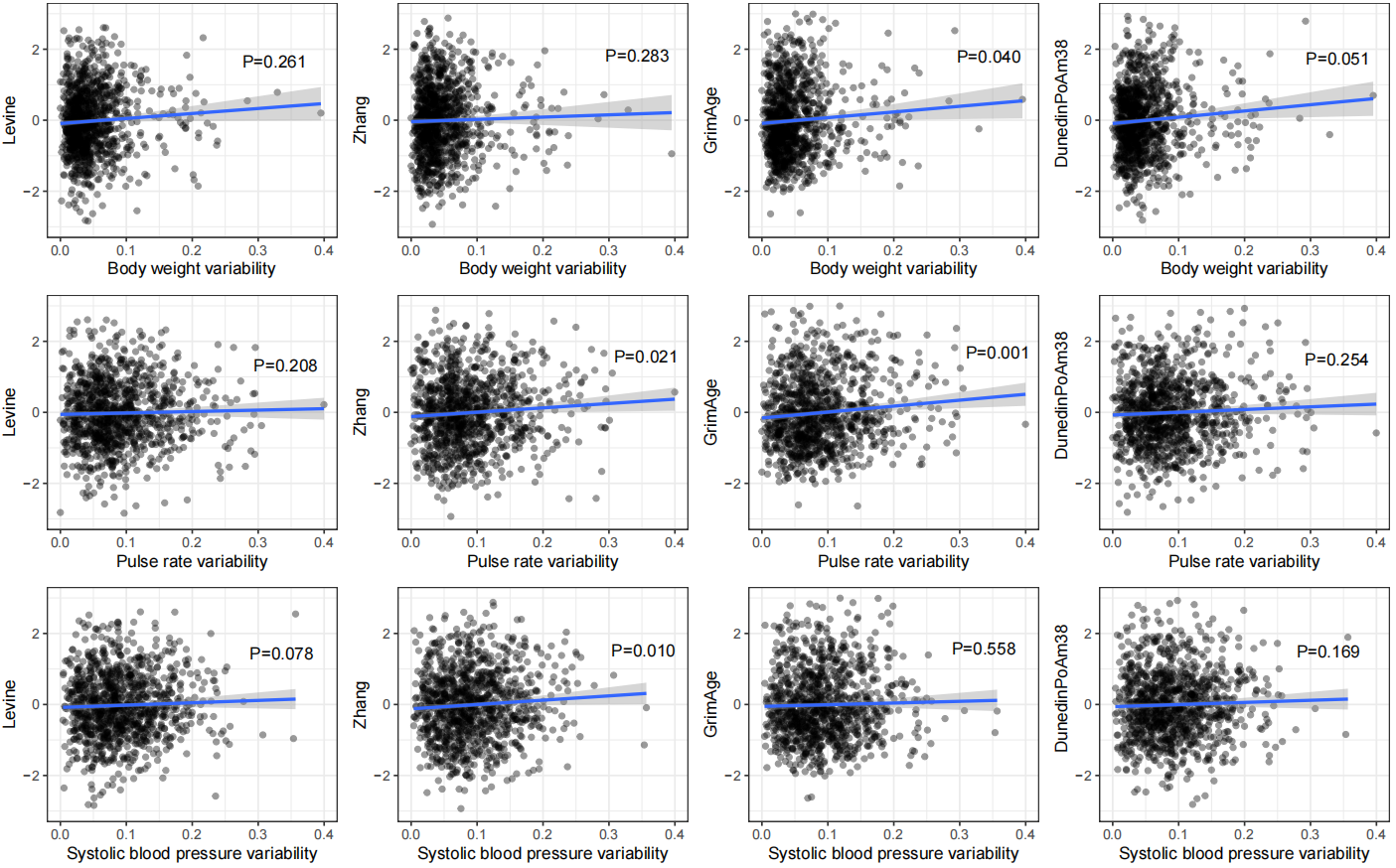


Univariate linear regression model was used to fit the lines. The y-axes were the epigenetic age acceleration represented by the standardized residuals of the clock values regressed on the baseline age.

# Table S1. Descriptive statistics of the 13 epigenetic clocks (N=1,047).

| **Epigenetic clocks** | **Generation** | **Mean** | **SD** | **Maximum** | **Minimum** |
| --- | --- | --- | --- | --- | --- |
| **Clocks** |  |  |  |  |  |
| Horvath 1 | 1 | 71.18 | 8.06 | 114.52 | 32.41 |
| Hannum | 1 | 60.63 | 7.16 | 107.79 | 34.50 |
| Horvath 2 | 1 | 75.64 | 6.30 | 97.16 | 43.62 |
| Lin | 1 | 64.69 | 9.74 | 133.27 | 7.44 |
| Weidner | 1 | 70.54 | 11.73 | 148.87 | 38.24 |
| VidalBralo | 1 | 66.60 | 5.72 | 97.11 | 36.47 |
| Yang | 1 | 0.07 | 0.02 | 0.23 | 0.03 |
| Bocklandt | 1 | 0.37 | 0.08 | 0.89 | 0.13 |
| Garagnani | 1 | 0.76 | 0.06 | 0.99 | 0.47 |
| Levine | 2 | 63.14 | 8.06 | 93.84 | 26.72 |
| Zhang | 2 | -0.98 | 0.43 | 0.51 | -2.27 |
| GrimAge | 2 | 73.66 | 6.25 | 90.93 | 51.34 |
| DunedinPoAm38 | 2 | 1.07 | 0.09 | 1.39 | 0.75 |
| **Epigenetic age acceleration** | | | | | |
| Horvath 1 | 1 | 0 | 1 | 6.94 | -4.82 |
| Hannum | 1 | 0 | 1 | 7.96 | -5.49 |
| Horvath 2 | 1 | 0 | 1 | 4.03 | -5.31 |
| Lin | 1 | 0 | 1 | 6.83 | -6.59 |
| Weidner | 1 | 0 | 1 | 6.26 | -3.05 |
| VidalBralo | 1 | 0 | 1 | 5.92 | -5.92 |
| Yang | 1 | 0 | 1 | 7.51 | -1.94 |
| Bocklandt | 1 | 0 | 1 | 6.88 | -3.34 |
| Garagnani | 1 | 0 | 1 | 4.19 | -5.37 |
| Levine | 2 | 0 | 1 | 4.19 | -4.09 |
| Zhang | 2 | 0 | 1 | 3.37 | -2.93 |
| GrimAge | 2 | 0 | 1 | 3.43 | -2.64 |
| DunedinPoAm38 | 2 | 0 | 1 | 3.43 | -3.57 |

Each clock value was regressed on age and the residual thus reflect the epigenetic age acceleration. To allow for comparison across different clocks, we used student standardized residuals, and a higher value indicated faster biological aging.

# Table S2. Associations of the composite score of variability in physiological measures with mortality in the HRS and CHARLS with different adjustments.

|  | **Cases/Person-years** | **Hazard ratio^*^ (95% Confidence Interval)** | | |
| --- | --- | --- | --- | --- |
|  |  | **Model 1** | **Model 2** | **Model 3** |
| **HRS** |  |  |  |  |
| 0 point | 107/6748 | 1 (Ref.) | 1 (Ref.) | 1 (Ref.) |
| 1 point | 171/8176 | 1.28 [1.00, 1.63] | 1.15 [0.90, 1.47] | 1.13 [0.89, 1.45] |
| 2 points | 143/4629 | 1.64 [1.28, 2.12] | 1.42 [1.10, 1.83] | 1.39 [1.08, 1.80] |
| 3 points | 62/943 | 3.44 [2.51, 4.72] | 2.69 [1.94, 3.72] | 2.66 [1.93, 3.69] |
| 1-point increment |  | 1.43 [1.29, 1.58] | 1.32 [1.19, 1.46] | 1.32 [1.19, 1.46] |
| P-trend |  | <0.001 | <0.001 | <0.001 |
| **CHARLS** |  |  |  |  |
| 0 point | 68/6551 | 1 (Ref.) | 1 (Ref.) | 1 (Ref.) |
| 1 point | 112/8558 | 1.17 [0.87, 1.59] | 1.16 [0.86, 1.57] | 1.10 [0.81, 1.49] |
| 2 points | 87/4595 | 1.53 [1.11, 2.10] | 1.48 [1.08, 2.04] | 1.35 [0.98, 1.86] |
| 3 points | 28/977 | 2.15 [1.38, 3.35] | 2.13 [1.37, 3.32] | 1.93 [1.24, 3.02] |
| 1-point increment |  | 1.28 [1.12, 1.45] | 1.26 [1.11, 1.44] | 1.22 [1.07, 1.38] |
| P-trend |  | <0.001 | <0.001 | 0.003 |
| **Pooled** |  |  |  |  |
| 1-point increment |  | 1.37 [1.26, 1.48] | 1.30 [1.20, 1.41] | 1.27 [1.18, 1.39] |
| P-trend |  | <0.001 | <0.001 | <0.001 |
| P-heterogeneity |  | 0.186 | 0.582 | 0.344 |

HRS, Health and Retirement Study; CHARLS, China Health and Retirement Longitudinal Study.

**^*^** Model 1 was adjusted for age (continuous) and gender (female or male). Model 2 was further adjusted for education (high school degree, yes / no), marriage status (yes / no), residence (rural / urban) in the CHARLS, race (White / Black / Others) in the HRS, drinking status (current / ever / never), smoking status (current / ever / never), physical activity (>1 time per week / 1-3 times per month / never in the HRS and any / never / missing in the CHARLS), and household income (quartiles in the HRS and ≤9999 yuan / ≥10000 yuan / missing in the CHARLS). Model 3 was further adjusted for body mass index, systolic blood pressure, and pulse rate (all continuous) in 2014/2016 (HRS) or in 2015 (CHARLS).

# Table S3. Associations between each one-point increment in the composite score of variability and epigenetic age acceleration indicated by 13 first- and second-generation epigenetic clocks.

| **EAA** | **Generation** | **β^*^ (95% CI)** | **Adjusted P-value^†^** |
| --- | --- | --- | --- |
| Horvath 1 | 1 | 0.051 (-0.020, 0.122) | 0.295 |
| Hannum | 1 | **0.103 (0.034, 0.172)** | 0.016 |
| Horvath 2 | 1 | 0.045 (-0.025, 0.116) | 0.341 |
| Lin | 1 | 0.040 (-0.031, 0.111) | 0.356 |
| Weidner | 1 | 0.015 (-0.057, 0.086) | 0.749 |
| VidalBralo | 1 | 0.059 (-0.011, 0.128) | 0.212 |
| Yang | 1 | 0.039 (-0.031, 0.109) | 0.356 |
| Bocklandt | 1 | -0.004 (-0.074, 0.066) | 0.911 |
| Garagnani | 1 | 0.036 (-0.035, 0.107) | 0.382 |
| Levine | 2 | **0.126 (0.055, 0.196)** | <0.001 |
| Zhang | 2 | **0.094 (0.028, 0.160)** | 0.016 |
| GrimAge | 2 | **0.096 (0.040, 0.153)** | 0.007 |
| DunedinPoAm38 | 2 | 0.074 (0.009, 0.139) | 0.068 |

EAA: epigenetic age acceleration

**^*^**β-coefficients were derived from models adjusted for age (continuous), gender (female or male, unless in gender-stratified analyses), education (high school degree, yes / no), marriage status (yes / no), race (White / Black / Others), drinking status (current / ever / never), smoking status (current / ever / never), physical activity (>1 time per week / 1-3 times per month / never), household income (in quartiles), body mass index, systolic blood pressure, and pulse rate (all continuous) in 2014/2016.

^†^P-values were adjusted using Benjamini-Hochberg methods.

# Table S4. Associations of BWV, PRV and SBPV with epigenetic age acceleration indicated by 13 first- and second-generation epigenetic clocks.

| **EAA** | **BWV (per SD)** | **PRV (per SD)** | **SBPV (per SD)** |
| --- | --- | --- | --- |
|  | β***** (95% CI) | β***** (95% CI) | β***** (95% CI) |
| **Model 1** |  |  |  |
| Horvath 1 | 0.009 (-0.055, 0.072) | 0.009 (-0.052, 0.070) | 0.071 (0.008, 0.133) |
| Hannum | 0.030 (-0.033, 0.093) | 0.053 (-0.008, 0.114) | 0.045 (-0.018, 0.108) |
| Horvath 2 | 0.044 (-0.019, 0.107) | 0.024 (-0.037, 0.085) | 0.017 (-0.046, 0.080) |
| Lin | -0.004 (-0.068, 0.059) | 0.016 (-0.045, 0.076) | 0.031 (-0.032, 0.094) |
| Weidner | -0.037 (-0.100, 0.026) | 0.046 (-0.015, 0.107) | 0.012 (-0.051, 0.075) |
| VidalBralo | 0.014 (-0.049, 0.077) | 0.058 (-0.003, 0.119) | 0.044 (-0.019, 0.106) |
| Yang | 0.020 (-0.043, 0.084) | 0.046 (-0.014, 0.107) | 0.021 (-0.042, 0.084) |
| Bocklandt | 0.025 (-0.038, 0.089) | 0.003 (-0.058, 0.064) | -0.014 (-0.077, 0.049) |
| Garagnani | 0.034 (-0.029, 0.097) | 0.017 (-0.044, 0.078) | 0.028 (-0.035, 0.091) |
| Levine | 0.036 (-0.027, 0.099) | 0.039 (-0.022, 0.100) | 0.056 (-0.006, 0.119) |
| Zhang | 0.035 (-0.029, 0.098) | 0.072 (0.011, 0.132) | 0.082 (0.019, 0.145) |
| GrimAge | 0.066 (0.003, 0.129) | 0.103 (0.042, 0.164) | 0.019 (-0.044, 0.082) |
| DunedinPoAm38 | 0.063 (-0.000, 0.126) | 0.035 (-0.025, 0.096) | 0.044 (-0.019, 0.107) |
| **Model 2** |  |  |  |
| Horvath 1 | 0.016 (-0.047, 0.079) | -0.002 (-0.063, 0.059) | 0.080 (0.017, 0.142) |
| Hannum | 0.043 (-0.019, 0.105) | 0.035 (-0.026, 0.095) | 0.060 (-0.001, 0.122) |
| Horvath 2 | 0.053 (-0.010, 0.116) | 0.011 (-0.050, 0.072) | 0.027 (-0.036, 0.090) |
| Lin | 0.002 (-0.061, 0.065) | 0.006 (-0.055, 0.067) | 0.039 (-0.024, 0.102) |
| Weidner | -0.034 (-0.097, 0.030) | 0.042 (-0.020, 0.103) | 0.016 (-0.048, 0.079) |
| VidalBralo | 0.026 (-0.037, 0.088) | 0.041 (-0.019, 0.101) | 0.058 (-0.005, 0.120) |
| Yang | 0.018 (-0.045, 0.082) | 0.050 (-0.011, 0.111) | 0.019 (-0.044, 0.082) |
| Bocklandt | 0.015 (-0.047, 0.078) | 0.018 (-0.042, 0.079) | -0.026 (-0.089, 0.036) |
| Garagnani | 0.032 (-0.031, 0.096) | 0.020 (-0.041, 0.081) | 0.026 (-0.037, 0.089) |
| Levine | 0.042 (-0.021, 0.106) | 0.031 (-0.030, 0.092) | 0.064 (0.001, 0.127) |
| Zhang | 0.049 (-0.013, 0.111) | 0.050 (-0.009, 0.110) | 0.100 (0.038, 0.161) |
| GrimAge | 0.090 (0.031, 0.150) | 0.068 (0.011, 0.125) | 0.047 (-0.012, 0.106) |
| DunedinPoAm38 | 0.069 (0.006, 0.133) | 0.027 (-0.034, 0.088) | 0.052 (-0.011, 0.114) |
| **Model 3** |  |  |  |
| Horvath 1 | 0.018 (-0.046, 0.082) | 0.002 (-0.059, 0.063) | 0.084 (0.020, 0.147) |
| Hannum | 0.049 (-0.013, 0.111) | 0.032 (-0.028, 0.092) | 0.054 (-0.008, 0.116) |
| Horvath 2 | 0.055 (-0.008, 0.118) | 0.013 (-0.048, 0.074) | 0.022 (-0.041, 0.085) |
| Lin | 0.006 (-0.059, 0.070) | 0.003 (-0.058, 0.064) | 0.037 (-0.026, 0.101) |
| Weidner | -0.028 (-0.092, 0.037) | 0.041 (-0.021, 0.102) | 0.018 (-0.046, 0.082) |
| VidalBralo | 0.029 (-0.034, 0.091) | 0.035 (-0.025, 0.095) | 0.059 (-0.003, 0.121) |
| Yang | 0.006 (-0.057, 0.068) | 0.054 (-0.006, 0.115) | 0.009 (-0.053, 0.072) |
| Bocklandt | 0.004 (-0.058, 0.067) | 0.019 (-0.041, 0.079) | -0.031 (-0.093, 0.031) |
| Garagnani | 0.026 (-0.038, 0.090) | 0.022 (-0.040, 0.083) | 0.023 (-0.041, 0.086) |
| Levine | 0.030 (-0.033, 0.094) | 0.023 (-0.038, 0.084) | 0.053 (-0.010, 0.116) |
| Zhang | 0.016 (-0.044, 0.076) | 0.032 (-0.025, 0.089) | 0.079 (0.020, 0.138) |
| GrimAge | 0.041 (-0.010, 0.092) | 0.055 (0.005, 0.104) | 0.024 (-0.026, 0.075) |
| DunedinPoAm38 | 0.033 (-0.026, 0.091) | 0.019 (-0.037, 0.075) | 0.039 (-0.019, 0.098) |

BWV: body weight variability; PRV: pulse rate variability; SBPV: systolic blood pressure variability; EAA: epigenetic age acceleration

*Model 1 was univariate model with no covariates adjusted. Model 2 was adjusted for age (continuous) and gender (male / female). Model 3 was adjusted for age (continuous), gender (male / female), education (high school degree, yes / no), marriage status (yes / no), race (White / Black / Others), drinking status (current / ever / never), smoking status (current / ever / never), physical activity (>1 time per week / 1-3 times per month / never), household income (in quartiles), body mass index, systolic blood pressure, and pulse rate (all continuous) in 2014/2016.

# Table S5. Sensitivity analyses for the associations between each one-point increment in the composite score of variability with risk of all-cause mortality.

| **Sensitivity analyses*** | **CHARLS** | **HRS** | **Pooled HR**^‡^ | **P-heterogeneity** |
| --- | --- | --- | --- | --- |
|  | **HR [95% CI]** | **HR [95% CI]** |  |  |
| Sensitivity analysis 1 | 1.26 [1.10, 1.44] | 1.27 [1.15, 1.41] | 1.27 [1.16, 1.38] | 0.927 |
| Sensitivity analysis 2-SD | 1.28 [1.12, 1.46] | 1.32 [1.19, 1.46] | 1.31 [1.21, 1.42] | 0.366 |
| Sensitivity analysis 2-VIM^†^ | 1.23 [1.07, 1.41] | 1.32 [1.19, 1.47] | 1.28 [1.19, 1.40] | 0.719 |
| Sensitivity analysis 3 | 1.25 [1.10, 1.43] | 1.35 [1.21, 1.49] | 1.31 [1.21, 1.42] | 0.426 |
| Sensitivity analysis 4 | 1.18 [1.03, 1.35] | 1.24 [1.12, 1.37] | 1.22 [1.13, 1.32] | 0.564 |
| Sensitivity analysis 5 | 1.11 [1.02, 1.20] | 1.15 [1.08, 1.22] | 1.14 [1.08, 1.20] | 0.495 |
| Sensitivity analysis 6 | NA | NA | NA | NA |

CHARLS: China Health and Retirement Longitudinal Study; HRS: Health and Retirement Study; HR: Hazard Ratio; CI: confidence interval

*Sensitivity analysis 1: further adjusted for health conditions including diabetes mellitus, heart disease, and stroke. Sensitivity analysis 2: using standard deviation (SD) and variation independent of the mean (VIM) in place of the coefficient of variation Sensitivity analysis 3: using diastolic blood pressure variability (DBPV) in place of SBPV. Sensitivity analysis 4: redefined high-variability of BWV, PRV, and BPV as values above medians rather than the top tertiles. Sensitivity 5: alternatively assigned 0, 1, 2 to increasing tertiles of variability indicators to construct a new composite score ranging from 0 to 6. Sensitivity analysis 6: further adjusted for cell-type proportions using results from a white blood cell (WBC) differential assay (ignored).

^†^VIM was calculated as the SD divided by the mean to the power x and multiplied by the population mean to the power x. The power x is obtained by fitting a curve through a plot of SD against mean using the model SD=$a\times{mean}^{x}$, where x was derived by nonlinear regression analysis.

^‡^HRs were pooled using fixed effect models.

# Table S6. Sensitivity analyses for the associations between variability of physiological measures and epigenetic age acceleration

| **Epigenetic age acceleration** | **BWV (per SD)** | **PRV (per SD)** | **BPV (per SD)** | **The composite score (per one-point increment)** |
| --- | --- | --- | --- | --- |
|  | β (95% CI) ^b^ | β (95% CI) ^b^ | β (95% CI) ^b^ | β (95% CI) ^c^ |
| **Further adjusted for health conditions*** | |  |  |  |
| Levine | 0.027 (-0.036, 0.090) | 0.009 (-0.052, 0.070) | 0.046 (-0.016, 0.108) | 0.110 (0.039, 0.180) |
| Zhang | 0.015 (-0.045, 0.074) | 0.014 (-0.044, 0.071) | 0.074 (0.015, 0.132) | 0.074 (0.008, 0.140) |
| GrimAge | 0.043 (-0.008, 0.095) | 0.038 (-0.012, 0.088) | 0.013 (-0.038, 0.064) | 0.081 (0.023, 0.138) |
| DunedinPoAm38 | 0.037 (-0.022, 0.097) | 0.003 (-0.055, 0.060) | 0.028 (-0.031, 0.086) | 0.060 (-0.006, 0.127) |
| **Further adjusted for cell-type proportions using results from a white blood cell (WBC) differential assay ^†^** | | | | |
| Levine | 0.027 (-0.034, 0.089) | 0.027 (-0.032, 0.087) | 0.044 (-0.017, 0.105) | 0.117 (0.049, 0.186) |
| Zhang | 0.010 (-0.045, 0.066) | 0.034 (-0.020, 0.088) | 0.066 (0.011, 0.121) | 0.079 (0.017, 0.141) |
| GrimAge | 0.038 (-0.011, 0.086) | 0.059 (0.012, 0.106) | 0.011 (-0.038, 0.059) | 0.084 (0.030, 0.138) |
| DunedinPoAm38 | 0.024 (-0.029, 0.077) | 0.017 (-0.034, 0.067) | 0.020 (-0.033, 0.072) | 0.049 (-0.010, 0.108) |
| **Using standard deviation (SD) in place of coefficient of variation (per SD)** | | |  |  |
| Levine | 0.025 (-0.038, 0.087) | 0.031 (-0.031, 0.093) | 0.053 (-0.009, 0.115) | 0.097 (0.025, 0.169) |
| Zhang | 0.004 (-0.055, 0.063) | 0.036 (-0.022, 0.094) | 0.085 (0.027, 0.143) | 0.092 (0.025, 0.160) |
| GrimAge | 0.030 (-0.020, 0.080) | 0.065 (0.015, 0.115) | 0.025 (-0.025, 0.075) | 0.104 (0.046, 0.162) |
| DunedinPoAm38 | 0.033 (-0.024, 0.090) | 0.028 (-0.029, 0.085) | 0.036 (-0.020, 0.093) | 0.060 (-0.007, 0.127) |
| **Using variation independent of the mean (VIM) in place of coefficient of variation (per SD) ^‡^** | | | |  |
| Levine | 0.030 (-0.032, 0.091) | 0.020 (-0.040, 0.081) | 0.053 (-0.008, 0.114) | 0.126 (0.056, 0.197) |
| Zhang | 0.020 (-0.038, 0.078) | 0.030 (-0.027, 0.087) | 0.077 (0.020, 0.134) | 0.093 (0.027, 0.159) |
| GrimAge | 0.043 (-0.006, 0.092) | 0.052 (0.003, 0.101) | 0.024 (-0.025, 0.072) | 0.100 (0.043, 0.156) |
| DunedinPoAm38 | 0.032 (-0.024, 0.089) | 0.017 (-0.039, 0.073) | 0.040 (-0.016, 0.096) | 0.079 (0.013, 0.144) |
| **Using diastolic blood pressure variability (DBPV) in place of SBPV ^§^** | | |  |  |
| Levine |  |  | 0.062 (-0.003, 0.127) | 0.119 (0.048, 0.190) |
| Zhang |  |  | 0.070 (0.010, 0.131) | 0.083 (0.016, 0.150) |
| GrimAge |  |  | 0.007 (-0.045, 0.060) | 0.080 (0.022, 0.139) |
| DunedinPoAm38 |  |  | 0.012 (-0.048, 0.072) | 0.032 (-0.035, 0.099) |
| **Redefined the high-variability measures using the dichotomized indicators of value above medians ^\|\|^** | | | |  |
| Levine |  |  |  | 0.085 (0.018, 0.151) |
| Zhang |  |  |  | 0.091 (0.029, 0.153) |
| GrimAge |  |  |  | 0.100 (0.047, 0.154) |
| DunedinPoAm38 |  |  |  | 0.044 (-0.017, 0.106) |
| **Alternatively assigned 0, 1, 2 to increasing tertiles of variability indicators, and the composite score ranged from 0-6 points) ^\|\|^** | | | | |
| Levine |  |  |  | 0.058 (0.017, 0.099) |
| Zhang |  |  |  | 0.048 (0.009, 0.086) |
| GrimAge |  |  |  | 0.048 (0.014, 0.082) |
| DunedinPoAm38 |  |  |  | 0.034 (-0.004, 0.073) |

BWV: body weight variability; PRV: pulse rate variability; BPV: blood pressure variability

^*^Health conditions included diabetes mellitus, heart disease, and stroke. Models were adjusted for age (continuous), gender (male / female), education (high school degree, yes / no), marriage status (yes / no), race (White / Black / Others), drinking status (current / ever / never), smoking status (current / ever / never), physical activity (>1 time per week / 1-3 times per month / never), household income (in quartiles), body mass index, systolic blood pressure, pulse rate (all continuous) and health conditions in 2014/2016.

^†^ Models were adjusted for age (continuous), gender (male / female), education (lower than high school or not), marriage status (yes / no), race (White / Black / Others), drinking status (current / ever / never), smoking status (current / ever / never), physical activity (>1 time per week / 1-3 times per month / never), household income (in quartiles), body mass index, systolic blood pressure, pulse rate (all continuous), and cell-type proportions.

^‡^ VIM was calculated as the SD divided by the mean to the power x and multiplied by the population mean to the power x. The power x is obtained by fitting a curve through a plot of SD against mean using the model SD=$a\times{mean}^{x}$, where x was derived by nonlinear regression analysis.

^§^ The composite score was the sum of the three scores of BWV, PRV, and DBPV and ranged from 0 to 3 points. β-coefficients were derived from models adjusted for age (continuous), gender (male / female), education (high school degree, yes / no), marriage status (yes / no), race (White / Black / Others), drinking status (current / ever / never), smoking status (current / ever / never), physical activity (>1 time per week / 1-3 times per month / never), household income (in quartiles), body mass index, diastolic blood pressure, pulse rate (all continuous)

^||^ β-coefficients were derived from models adjusted for age (continuous), gender (male / female), education (high school degree, yes / no), marriage status (yes / no), race (White / Black / Others), drinking status (current / ever / never), smoking status (current / ever / never), physical activity (>1 time per week / 1-3 times per month / never), household income (in quartiles), body mass index, systolic blood pressure, and pulse rate (all continuous) in 2014/2016.
